# Inhibitory Effects of the Polyphenols from the Root of *Rhizophora apiculata* Blume on Fatty Acid Synthase Activity and Human Colon Cancer Cells

**DOI:** 10.3390/molecules29051180

**Published:** 2024-03-06

**Authors:** Yan Liang, Yue Ban, Lei Liu, Yanchun Li

**Affiliations:** 1 School of Sports Sciences, Beijing Sport University, No. 48, Xinxi Road, Beijing 100084, China; yanliang@cupes.edu.cn; 2 School of Kinesiology and Health, Capital University of Physical Education and Sports, No. 11, Beisanhuanxi Road, Beijing 100191, China; banyue@cupes.edu.cn; 3 College of Chemistry and Materials Engineering, Beijing Technology & Business University (BTBU), Beijing 100048, China

**Keywords:** fatty acid synthase, inhibitor, human colon cancer, β-ketoacyl reductase, *Rhizophora apiculata* Blume, polyphenol

## Abstract

Marine mangrove vegetation has been traditionally employed in folk medicine to address various ailments. Notably, *Rhizophora apiculata* Blume has exhibited noteworthy properties, demonstrating efficacy against cancer, viruses, and bacteria. The enzyme fatty acid synthase (FAS) plays a pivotal role in de novo fatty acid synthesis, making it a promising target for combating colon cancer. Our study focused on evaluating the FAS inhibitory effects of both the crude extract and three isolated compounds from *R. apiculata*. The n-butanol fraction of *R. apiculata* extract (BFR) demonstrated a significant inhibition of FAS, with an IC_50_ value of 93.0 µg/mL. For inhibition via lyoniresinol-3α-*O*-β-rhamnopyranoside (LR), the corresponding IC_50_ value was 20.1 µg/mL (35.5 µM). LR competitively inhibited the FAS reaction with acetyl-CoA, noncompetitively with malonyl-CoA, and in a mixed manner with NADPH. Our results also suggest that both BFR and LR reversibly bind to the KR domain of FAS, hindering the reduction of saturated acyl groups in fatty acid synthesis. Furthermore, BFR and LR displayed time-dependent inhibition for FAS, with k_obs_ values of 0.0045 min^−1^ and 0.026 min^−1^, respectively. LR also exhibited time-dependent inhibition on the KR domain, with a k_obs_ value of 0.019 min^−1^. In human colon cancer cells, LR demonstrated the ability to reduce viability and inhibit intracellular FAS activity. Notably, the effects of LR on human colon cancer cells could be reversed with the end product of FAS-catalyzed chemical reactions, affirming the specificity of LR on FAS. These findings underscore the potential of BFR and LR as potent FAS inhibitors, presenting novel avenues for the treatment of human colon cancer.

## 1. Introduction

Colon cancer ranks among the most prevalent cancers globally, particularly in Western nations. Numerous epidemiological studies suggest a potential correlation between the consumption of Western-style diets, rich in elements such as red meat, and an elevated incidence of colon cancer [1]. In 2019, approximately 2.2 million new cases of colorectal and anal cancer were diagnosed worldwide, resulting in approximately 1.1 million deaths [2]. Across both genders, the global incidence of these cancers exhibited an upward trend from 1990 to 2019, while the mortality trend showed a decline during the same period [2]. The disease poses substantial risks, ranging from localized complications within the colon to the potential for metastasis, which can significantly diminish survival rates [3]. To address this challenge, ongoing research has been dedicated to exploring novel drugs and therapeutic approaches for the treatment of colon cancer. Investigational compounds, both synthetic and derived from natural sources, are being studied for their potential efficacy in inhibiting key molecular pathways involved in cancer progression. These advancements in drug research offer promising avenues for developing more targeted and effective treatments, bringing hope for improved outcomes and quality of life for individuals affected by colon cancer [4,5,6].

Obesity is becoming a worldwide epidemic that is being increasingly recognized as a worsening factor in a number of chronic diseases, including colon cancer [7]. A large body of scientific literature indicates that obese people are prone to a variety of cancers, especially colorectal cancer [8,9]. Fatty acid is an important source of fat synthesis, but excess of their ectopic accumulation in other functional organs will lead to obesity-related diseases [10]. The de novo synthesis of long chain fatty acids is catalyzed by fatty acid synthase (FAS, EC 2.3.1.85), which has been considered as an antiobesity and anticancer target recently [11,12].

Within the confines of animal organisms, FAS facilitates the production of extended saturated fatty acids from acetyl-CoA, malonyl-CoA, and nicotinamide adenine dinucleotide phosphate (NADPH) through the stepwise operation of its seven operational units [13,14]. FAS is constituted of two matching components (260–270 kDa), each harboring an acyl carrier protein (ACP) alongside six enzymatic centers, including acetyl/malonyl transferase (AMT), β-ketoacyl synthase (KS), β-ketoacyl reductase (KR), β-hydroxyacyl dehydratase (DH), enoyl reductase (ER), and thioesterase (TE) [15]. In instances of genetic obesity in rats and diabetes in humans, there is notable upregulation of FAS in adipose tissue [16,17,18]. Investigations have indicated that administering FAS inhibitors to mice resulted in decreased appetite and significant weight reduction. These inhibitors suppressed the expression of the hunger-inducing signal neuropeptide Y, a process likely mediated by malonyl-CoA. Consequently, FAS potentially serves as a pivotal regulator in the control of feeding behavior [19]. Considering the findings from the aforementioned studies, it is evident that FAS inhibitors offer promising prospects for weight loss therapy. However, the current arsenal of FAS inhibitors remains limited. 

Mangroves, an ecologically related group of plant species distributed along tropical and subtropical coastlines around the world, provide a unique ecosystem and play several important biological and ecological roles [20]. *Rhizophora apiculata* Blume is a species of mangrove in the Rhizophoraceae family. It is found in Australia, Guam, India, Indonesia, Malaysia, Micronesia, New Caledonia, Papua New Guinea, the Philippines, Singapore, the Solomon Islands, Sri Lanka, the Maldives, Thailand, Vanuatu, Vietnam, and south of China. Phytochemical studies have revealed that *R. apiculata* contains lignans, polysaccharides, catechins, diterpenoids, and triterpenoids [21]. *R. apiculata* exerts numerous biological activities and health-promoting properties, such as antibacterial [22], antitumor [21], antivirus [23], and antioxidant activity [24]. Recently, we have undertaken a screening of hundreds of plant extracts against FAS, among which we discovered that the crude extract of the stems of *R. apiculata* has obvious inhibitive effects on FAS.

To the best of our knowledge, the effects of the *R. apiculata* extract and its active compounds on FAS activity have not been studied. Therefore, we designed the experiments in the current study to confirm the inhibitory effects of *R. apiculata* extract on FAS. We demonstrated, for the first time, that the n-butanol fraction of *R. apiculata* extract (BFR) and lyoniresinol-3α-*O*-β-rhamnopyranoside (LR) potently inhibited the activity of FAS. These results might reveal the health care function of *R. apiculata* from a novel point of view.

## 2. Results

### 2.1. Preparation of R. apiculata Extract and Pure Compounds

**Figure 1:**
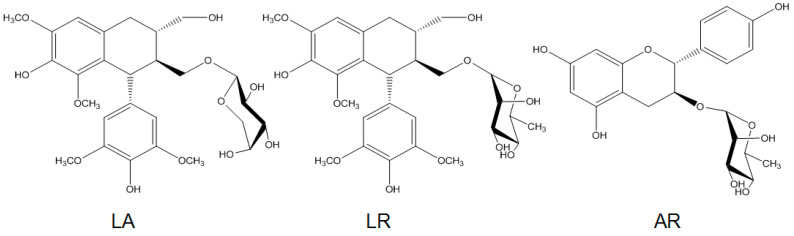
Chemical structures of LA (lyoniresinol-3α-*O*-β-arabinopyranoside), LR (lyoniresinol-3α-*O*-β-rhamnopyranoside), and AR (afzelechin-3-rhamnopyranoside).

Air-dried stems of *R. apiculata* (1.5 kg) were refluxed twice with 3 L of ethanol for 3 h. The solvent was evaporated in vacuo, and the crude extract was dissolved in H_2_O and partitioned with ethyl acetate (EtOAc) and n-butanol sequentially to yield three fractions. The yields of EtOAc fraction, n-butanol fraction, and water fraction were 12.0, 21.8, and 47 g, respectively. n-butanol fraction was subjected to macrospore resin column chromatography and further separated with preparative HPLC. Lyoniresinol-3α-*O*-β-arabinopyranoside (LA, 85 mg), LR (60 mg), and afzelechin-3-rhamnopyranoside (AR, 17 mg) were obtained after evaporating the solvent (Figure 1).

### 2.2. The Inhibition of FAS Activity by Different Fractions of R. apiculata Extract

Three fractions (EtOAc, *n*-butanol, and water) of *R. apiculata* were tested to determine their inhibitory activities on FAS. The n-butanol fraction showed inhibitory activity on FAS with IC_50_ of 93.0 ± 3.3 μg/mL (Table 1). However, EtOAc and water fractions showed no inhibitory effect. 

### 2.3. Inhibition of Overall Reaction and KR Reaction of FAS by BFR and LR

**Figure 2:**
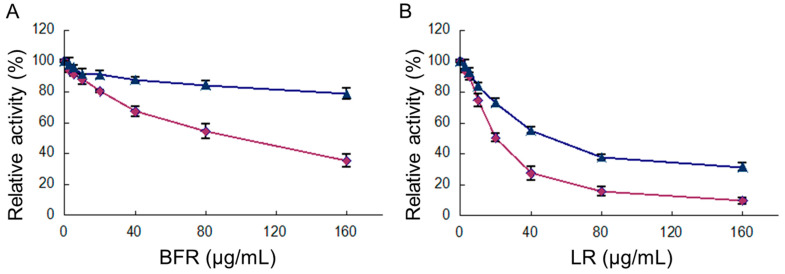
Evaluation of the FAS activity for fast-binding inhibition by BFR (n-butanol fraction of *R. apiculata* extract) and LR was conducted. The overall reaction (◆) and KR reaction (▲) of FAS were measured across different concentrations of BFR (**A**) and LR (**B**). The reported values represent the mean ± SD based on triplicate determinations.

The FAS activity for the overall reaction and KR reaction was assayed to determine the inhibitory capabilities of BFR and three main compounds in this fraction. The results showed that both reactions were inhibited by BFR, with IC_50_ values of 93.0 and 220.3 µg/mL for the overall and KR reactions (Figure 2A), respectively. For inhibition by LR, the relevant IC_50_ values were 20.1 µg/mL and 31.9 µg/mL, respectively (Figure 2B). However, LA and AR did not show a significant inhibitory effect on FAS (Table 1). 

### 2.4. Time-Dependent Inhibition of Overall and KR Reactions of FAS by BFR and LR

**Figure 3:**
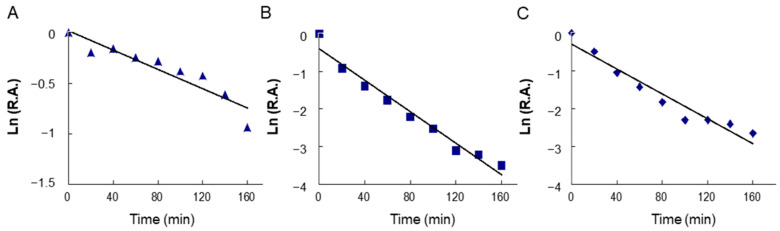
The time course of inhibition on FAS activities by BFR and LR is depicted. Specifically, the time-dependent inhibition of the overall reaction of FAS was measured in the presence of BFR (**A**) and LR (**B**). Additionally, (**C**) illustrates the inhibition of KR activity of FAS by LR. The FAS solution was mixed separately with BFR (3 mg/mL) or LR (3 mg/mL), and aliquots were assayed for relative activity at the indicated time intervals. R.A.—relative activity.

Figure 3 shows the time-dependent inhibition processes on FAS overall reactions by BFR (Figure 3A) and LR (Figure 3B) as well the KR reaction inhibited by LR (Figure 3C), respectively. All three reactions underwent a similar time-dependent inhibitory course. With a BFR concentration of 3.0 mg/mL, BFR exhibited inhibition of FAS with a k_obs_ value of 0.0047 min^−1^. With an LR concentration of 3.0 mg/mL, k_obs_ values were 0.021 and 0.017 min^−1^ for the overall reaction and KR, respectively. The less than 2-fold difference in the inactivation rate between overall reaction and KR suggested that the inhibition was mainly related to KR activity. These results showed LR was one of the active ingredients in *R. apiculata* when inhibiting FAS, and it reacted irreversibly with KR domain; thus, the time-dependent inhibition of BFR on FAS was mainly due to the reaction between LR and the KR domain of FAS.

### 2.5. Kinetics Studies of FAS Inhibition by LR

**Figure 4:**
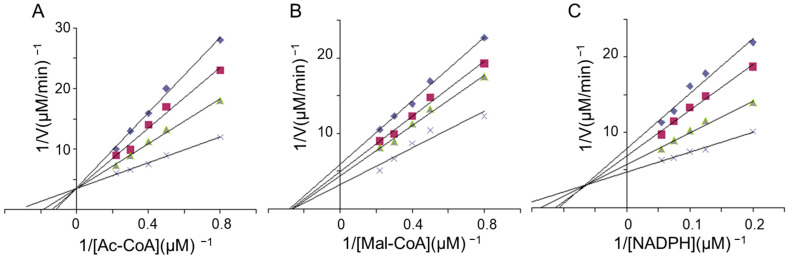
Lineweaver–Burke plots illustrating the inhibition of FAS activity by LR. Double reciprocal plots were generated for the inhibition of FAS by LR. In (**A**), the overall reaction of FAS was measured with acetyl-CoA as the variable substrate, and LR concentrations included 0 μg/mL (×), 5.0 μg/mL (▲), 10.0 μg/mL (■), and 15.0 μg/mL (◆). In (**B**), the overall reaction of FAS was measured with malonyl-CoA as the variable substrate and with LR concentrations as in (**A**). In (**C**), the KR activity was measured with NADPH as the variable substrate, using LR concentrations as in (**A**). Each data point represents the mean from 2–5 experiments.

The potential interference by LR at each substrate-binding site was determined kinetically. The double-reciprocal plots revealed that LR competitively inhibited FAS overall activity concerning acetyl-CoA (Figure 4A) and noncompetitively with respect to malonyl-CoA (Figure 4B). This finding suggests that LR might competitively bind to the acetyl-CoA binding site or the acetyl moiety site, distinct from where malonyl-CoA or its moiety binds. Furthermore, LR competitively inhibited the KR reaction of FAS concerning NADPH (Figure 4C), indicating that the NADPH binding site was one of LR’s multi-inhibitory targets. 

### 2.6. LR Reduced the Cell Viabilities of CaCo2, LoVo, and SW620 Cells

**Figure 5:**
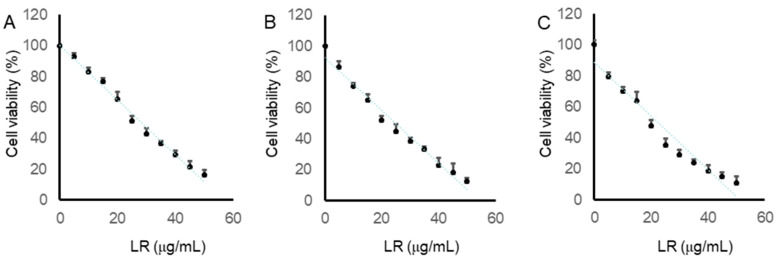
LR reduced the viability of CaCo2, LoVo, and SW620 cells. After a 24-h treatment with varying concentrations of LR, cell viabilities of CaCo2 cells (**A**), LoVo cells (**B**) and SW620 cells (**C**) were assessed using the MTT assay. The percentage of cell viability was calculated by comparing treated cells to control cells (0.1% DMSO). The presented data represent the mean ± SD from three independent experiments.

To assess LR’s cytotoxicity on human colon cancer cells, CaCo2, LoVo, and SW620 cells were exposed to varying concentrations (5, 10, 15, 20, 25, 30, 35, 40, 45, and 50 µg/mL) of LR for 24 h. Subsequently, cell viabilities were determined using the MTT assay. The dose-dependent decrease in the viability of the three colon cancer cell lines after 24 h of LR treatment is depicted in Figure 5. The half-maximal inhibitory concentration (IC_50_) values for LR in CaCo2, LoVo, and SW620 cells were found to be 28.4 µg/mL, 24.9 µg/mL, and 22.1 µg/mL, respectively. These results demonstrate LR’s ability to diminish colon cancer cell viability and suppress cell growth.

### 2.7. LR Inhibited the Intracellular FAS Activity in CaCo2, LoVo, and SW620 Cells

**Figure 6:**
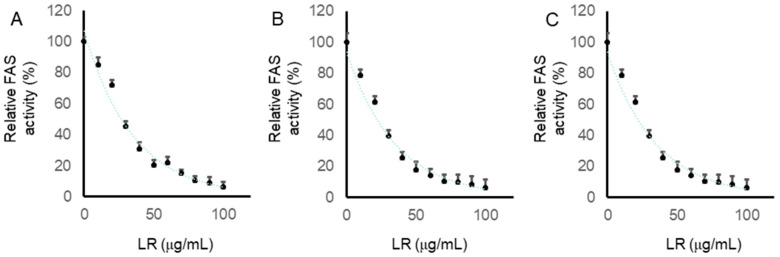
Inhibitory effect of LR on intracellular FAS activity in CaCo2, LoVo, and SW620 cells. Intracellular FAS activities in CaCo2 cells (**A**), LoVo cells (**B**), and SW620 cells (**C**) were assessed through spectrophotometry at 340 nm using NADPH. The relative FAS activities are presented as the means ±SD from three independent experiments with consistent outcomes.

We further checked the activity of FAS in cells treated with LR for 24 h, as described in the Material and Methods Section. Compared with the control, LR significantly inhibited the intracellular FAS activity in a dose-dependent manner. As shown in Figure 6, CaCo2, LoVo, and SW620 cells were treated with LR at concentrations of 0, 10, 20, 30, 40, 50, 60, 70, 80, 90, and 100 µg/mL for 24 h. Intracellular FAS activities in CaCo2, LoVo, and SW620 cells were all reduced in a dose-dependent manner. The IC_50_ values were 27.1, 25.5, and 21.8, respectively. In addition, the relative FAS activities were reduced to under 20% under treatment with LR at the concentration of 60 µg/mL in all the colon cancer cells. These results suggested that LR could inhibit intracellular FAS activity in CaCo2, LoVo, and SW620 cells.

### 2.8. Palmitic Acid Rescued the Reduction of Colon Cancer Cell Viability Induced by LR 

**Figure 7:**
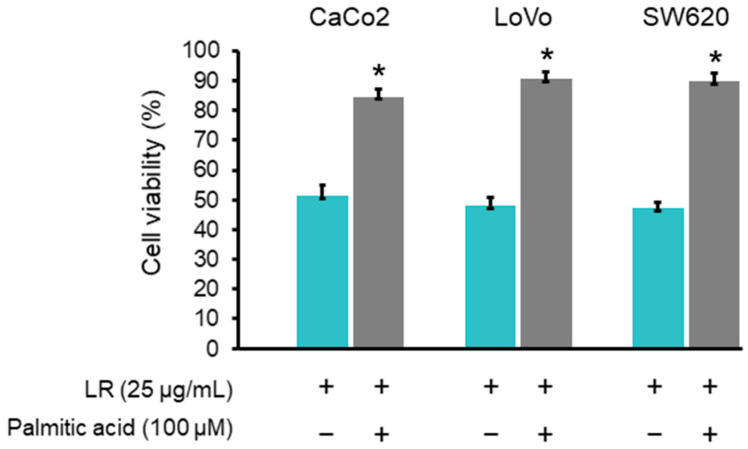
The effect of exogenous palmitic acid on the viability of LR-treated CaCo2, LoVo, and SW620 cells. The relative cell viability was determined with MTT assay. Data are expressed as the mean ± SD from three independent experiments with similar results. * *p* < 0.05 significantly different from respective control (LR treatment alone).

To confirm that the cell viability reduction induced by LR was related to the inhibition of FAS activity, CaCo2, LoVo, and SW620 cells were exposed for 24 h to LR at a concentration of 25 μg/mL in the presence of exogenous palmitic acid (100 μM), the end product of FAS reaction. The results showed that palmitic acid reduced the cytotoxic effects of LR, as the cell viabilities were restored significantly in CaCo2, LoVo, and SW620 cells (Figure 7).

## 3. Discussion

Varied levels of FAS expression between malignant and healthy cells have been proposed as a viable focus for the development of anticancer medications [25,26]. FAS expression has been documented in colorectal tumors, simultaneously to increase fatty acid synthesis [27,28]. There is a suggestion that pharmacologically inhibiting FAS diminishes cell proliferation and viability while prompting apoptosis in human cancerous cells [29]. Natural polyphenols exhibit the capability to inhibit FAS, offering a promising and well-tolerated avenue for colon cancer treatment [30,31].

Although several studies have elucidated the chemical components and biological effects of *R. apiculata*, none have explored its ability to inhibit FAS. In this study, we have shown that BFR and LR exhibit significant inhibitory effects on both the overall reaction and KR of FAS. Additionally, the inhibition of the overall reaction encompasses both reversible inhibition and slow-binding inactivation.

LR exhibits potent inhibition of the overall reaction of FAS, surpassing EGCG with a notably lower IC_50_ value. This robust inhibitory effect of LR on FAS highlights its promising potential as an anti-obesity treatment. 

In contrast to C75 but akin to EGCG and certain other polyphenols, LR predominantly targets the KR domain as a primary reaction site for FAS, inducing irreversible time-dependent inhibition. However, unlike EGCG, LR lacks the gallated ester’s carboxyl group, which has been identified as an active moiety in EGCG’s irreversible time-dependent inhibition mechanism [32]. Thus, the mode of action of LR on FAS differs from that of both C75 and EGCG.

Figure 3 illustrates the bi-phasic time-dependent inhibition of FAS by both BFR and LR, with comparable rate constants observed for the rapid phase. In contrast to other polyphenols like flavonoids and xanthones, which display potent fast-binding inhibition of FAS but lack time-dependent inhibition, LR presents markedly different kinetic outcomes [33]. Time-dependent inhibition generally indicates sustained and irreversible activity, which is essential for maintaining health. Inhibitors that exhibit time-dependent effects on FAS, such as resveratrol, C75, and EGCG, offer a notable advantage for potential in vivo applications.

Previous HPLC analysis revealed that LA, LR, and AR were the most abundant components in the n-butanol fraction of *R. apiculata* (0.068%, 0.066%, and 0.011%, respectively). These findings suggest that *R. apiculata* holds promise as a natural source of FAS inhibitors, with LR identified as the active compound. LR, the principal FAS inhibitor in *R. apiculata*, predominantly targets the KR domain of FAS. Kinetic analysis of LR indicates that its inhibition occurs at the site where NADPH binds to the KR domain, resembling the action of EGCG [33], which exhibits structural similarities with LR—both compounds feature two aromatic rings with specific spacing and hydroxyl substituents. Furthermore, owing to its smaller molecular structure, LR presents a weaker steric hindrance, possibly contributing to its comparatively stronger inhibition of FAS.

Although many polyphenols such as flavonoids, stilbenes, and xanthones have been found to have an inhibitory effect on FAS, only a few lignans with FAS-inhibition activity have been previously reported [34]. In this study, we examined the reversible inhibition on FAS by LA, another lignin, as well. Interestingly, LR can inhibit FAS, but LA cannot. LR, with a rhamnose ring connecting to lyoniresinol, exhibits high FAS inhibitory ability, whereas LA, possessing an arabinose sugar ring, shows much weaker inhibitory ability (IC_50_ > 200 μg/mL). Combined with previous findings indicating that avicularin, quercitrin, hyperoside, and isoquercitrin, four different quercetin glycosides, have different inhibitory activities on FAS [35], these observations suggest that the aglycone is not indispensable for inhibition against FAS, for which the sugar part of the glycoside substituent seems to be crucial.

Numerous studies on cell lines, animal models, and human epidemiological trials have shown the potential of polyphenols as anticancer agents [36,37]. Polyphenols have also been proven to affect cancer cell growth, cell cycle, and apoptosis [35,36,37]. Previous studies showed that the inhibition of FAS leads to a reduction in cancer cell viability [18]. Furthermore, although the reported FAS inhibitors, such as C75, cerulenin, α-mangostin, and resveratrol, have distinct structures, chemical properties, inhibitory mechanisms, and reaction sites on FAS, they all exhibit common effects: decreased viability in cancer cells [18,38,39]. Consequently, as an effective FAS inhibitor and with reduction effects on human colon cancer cells, LR has great potential for the clinical treatment of human cancers. 

While polyphenols offer numerous health benefits, their safety profile is complex and depends on various factors such as dose, bioavailability, interactions with medications, and individual sensitivity. When discussing the drug safety of polyphenols isolated form *R. apiculata*, several factors need to be considered. Many polyphenols have poor bioavailability, meaning they are not efficiently absorbed and metabolized in the body. This can limit their systemic effects but may also reduce the risk of adverse reactions. In the present study, we found that the effective concentration of LR is relatively low, indicating that its safety profile may be relatively high. In addition, LR demonstrated a clear dose-dependent relationship both for FAS inhibitory and cell viability assay, indicating its relatively high safety profile. As with any dietary component or supplement, it is essential to consume polyphenols in moderation and as part of a balanced diet to maximize their potential health benefits while minimizing any potential risks.

## 4. Materials and Methods 

### 4.1. Reagents

Acetyl-CoA, malonyl-CoA, NADPH, ethyl acetoacetate, palmitic acid, and epigallocatechin gallate (EGCG) were purchased from Sigma-Aldrich (St. Louis, MO, USA). All other reagents were local products with analytical grade purity.

### 4.2. Plant Source

The stems of *R. apiculata* were collected from the Hainan province of China and identified by Prof. Chuanchu Chen. A voucher specimen (FAS-2016) was deposited at the School of Kinesiology and Health, Capital University of Physical Education and Sports, Beijing, China.

### 4.3. Preparation of FAS and Its Substrates

The FAS employed in this study was sourced from chicken liver, given that the amino acid sequence of chicken FAS shares 63% identity with its human counterpart [40]. The chickens were purchased from Huadu Broiler Corporation, Beijing, China. The purification, storage, and application of the chicken liver FAS were conducted following established procedures [12]. Animal procedures adhered to the Guidelines for the Care and Use of Laboratory Animals as set forth by the Beijing Association for Laboratory Animal Science. The prepared enzyme exhibited homogeneity on polyacrylamide gel electrophoresis (PAGE) both in the presence and absence of SDS. Concentrations of the enzyme and substrates were determined through absorption measurements, with extinction coefficients being utilized as previously outlined [12].

### 4.4. Assay of FAS Activity 

The overall reaction of FAS and the β-ketoacyl reduction facilitated by KR (a functional domain of FAS) were assessed using an Amersham Pharmacia Ultrospec 4300 pro UV-Vis spectrophotometer (GE Healthcare China Company, Beijing, China) at 37 °C by monitoring the decrease of NADPH at 340 nm. The overall reaction mixture comprised potassium phosphate buffer (100 mM, pH 7.0), ethylenediaminetetraacetic acid (EDTA) (1 mM), 1,4-dithiothreitol (DTT) (1 mM), acetyl-CoA (6 µM), malonyl-CoA (12 µM), NADPH (37.5 µM), and chicken liver FAS (10 µg), with a total volume of 2.0 mL. For the KR reaction, the mixture included ethyl acetoacetate (40 mM), NADPH (35 µM), 1 mM of EDTA, and the enzyme (15 µg) in 100 mM of phosphate buffer at pH 7.0, with a total volume of 2.0 mL [41].

### 4.5. Assay of Fast-Binding Inhibition Activity

The inhibition kinetics of the inhibitor on FAS activity were determined by adding it to the reaction system before the initiation of the reaction, resulting in fast-binding inhibition that is reversible and typically caused by noncovalent binding to the enzyme. The concentration of ethanol in the reaction mixture did not exceed 0.2% (*v*/*v*), which had no significant impact on the FAS activity. The extent of inhibition was quantified by determining the IC_50_ value through a plot of residual activity against varying inhibitor concentrations.

### 4.6. Assay of Time-Dependent Inhibition Activity

The FAS solution was treated with various concentrations of inhibitors and incubated at 25 °C, with aliquots collected at specified time intervals to measure the remaining activity and to construct a time course profile. This type of inhibition is typically attributed to irreversible chemical reactions between the inhibitor and the enzyme, resulting in time-dependent inhibition. The rate constant for FAS inactivation was calculated from a semi-log plot of the time course through use of the formula Ln At/A0 = −k_obs_ t, where At/A0 represents the remaining activity at time t, and k_obs_ is the observed rate constant. Previous studies [39,40] have indicated that FAS activity inhibition occurs through both fast-binding and time-dependent mechanisms, with fast-binding reversible inhibition being occasionally insufficient to impact the enzyme.

### 4.7. Enzyme Kinetics Study

To investigate the potential interference of the inhibitor with each substrate binding site, the inhibitor concentration was fixed at various levels while one substrate concentration was incrementally increased while keeping the other substrate concentrations constant. Double reciprocal plots were generated for each inhibitor concentration to determine the competitive relationship between the variable substrate and inhibitor concentrations [32]. This study focused on examining fast-binding inhibition.

### 4.8. Cell Lines and Culture 

The human colon cancer cell lines CaCo2, LoVo, and SW620 were purchased from the Type Culture Collection of the Chinese Academy of Sciences (Shanghai, China). The CaCo2 cell line, originating from a human colon adenocarcinoma isolated in the 1970s, undergoes processes of proliferation, confluency, and differentiation during growth. When cultivated under standard conditions on semipermeable membranes, fully differentiated CaCo2 cells exhibit morphological characteristics similar to those of normal enterocytes. LoVo, another cell line isolated in 1971 from the large intestine of a 56-year-old White male with grade IV Dukes C colorectal cancer, offers applications in cancer, toxicology, and immuno-oncology research. SW620 cells, obtained from the large intestine of a 51-year-old male Dukes C colorectal cancer patient, are valuable resources for cancer and toxicology investigations. Cells were grown in Dulbecco’s Modified Eagle Medium (DMEM) supplemented with 10% fetal bovine serum (FBS) and were maintained in a humidified atmosphere containing 95% air and 5% CO_2_ at 37 °C. 

### 4.9. Cell Proliferation Inhibitory Activity Assay

LR was examined to evaluate its inhibition on the proliferation of CaCo2, LoVo, and SW620 cells. CaCo2, LoVo, and SW620 cells were grown in 96-well plates until reaching confluence and were treated with either DMSO (1:1000) or increasing doses of LR for 24 h at 37 °C. Following this, the cell medium was replaced with fresh medium containing 500 µg/mL MTT. After a 4-h incubation at 37 °C, the plates were decanted, and 150 µL of DMSO was added to dissolve the formazan crystals. Subsequently, the plate was analyzed using a microplate spectrophotometer (Multiskan, MK3) (Thermo Fisher Scientific Inc., Waltham, MA, USA) at a wavelength of 492 nm. Each experiment was conducted five times on three separate occasions.

### 4.10. Intracellular FAS Activity Assay

Cells were exposed to LR for 24 h, followed by collection through trypsinization, centrifugation, triple washing, and resuspension in cold PBS. Subsequently, the cells were sonicated at 4 °C and centrifuged at 12,000 rpm for 15 min at 4 °C to obtain particle-free supernatants. The intracellular FAS activity was assessed spectrophotometrically by measuring the reduction in absorbance at 340 nm due to the oxidation of NADPH. A total volume of 500 µL of buffer comprising 50 µL of particle-free supernatant, 25 mM of KH_2_PO_4_-K_2_HPO_4_ buffer, 0.25 mM of EDTA, 0.25 mM of dithiothreitol, 30 µM of acetyl-CoA, and 350 µM of NADPH (pH 7.0) was monitored at 340 nm for 1 min to establish baseline NADPH oxidation. Following the addition of 100 mM of malonyl-CoA, the reaction was further monitored for 1 min to determine FAS-dependent NADPH oxidation. 

### 4.11. Statistical Analysis

Data are expressed as the mean ± standard deviation (SD). One-way analysis of variance (ANOVA) followed by Tukey’s post hoc test was conducted using Origin 8.5 software (Originlab, Northampton, MS, USA) to identify statistical differences among three or more groups. Statistical significance was set at *p* < 0.05.

## 5. Conclusions

In an attempt to discover new potent FAS inhibitors, active compounds from *R. apiculata* were identified and evaluated. The results of the enzyme activity assay indicated that BFR and LR could inhibit FAS activity in both a reversible and irreversible manner. Kinetic results confirmed that the main active domain that BFR and LR acted on was KR. In addition, LR was found to have an inhibitory effect on intracellular FAS activity and could reduce the viability of human colon cancer cells significantly. The activity of LR could be rescued by the end product of a FAS-catalyzed chemical reaction, which proved the specificity of LR on FAS. Since many FAS inhibitors are reported to have the ability of treating human colon cancer, our results suggest that *R. apiculata* and LR can be considered to have the application potential in treatment of human colon cancer, and they may offer some ideas and clues for developing target-directed anticancer drugs for further in vivo studies. 

## Figures and Tables

**Table 1:** The inhibitory activity of the three fractions isolated from the stems of *R. apiculata* and three compounds against FAS ^a^.

Fractions/Compounds	FAS Inhibitory Activity IC_50_ (μg/mL)
EtOAc	>200 ^b^
*n*-butanol	93.0 ± 3.3
Water	N/I ^c^
LA	>200
LR	20.1 ± 1.6
AR	>200
EGCG ^d^	24.8 ± 2.2

^a^ IC_50_ values were determined via regression analyses and are expressed as the mean ± SD for three distinct experiments. ^b^ The level of IC_50_ value over 200 μg/mL indicated the weak activity against FAS. ^c^ N/I indicates that inhibitory activity could not be detected. ^d^ Positive control.

## Data Availability

Data are contained within the article.

## Abbreviations

The following abbreviations are used in this manuscript:

AR	afzelechin-3-rhamnopyranoside
BFR	n-butanol fraction of *R. apiculata* extract
DTT	1,4-dithiothreitol
DMEM	Dulbecco’s Modified Eagle Medium
EDTA	ethylenediaminetetraacetic acid
EGCG	epigallocatechin gallate
EtOAc	ethyl acetate
FAS	fatty acid synthase
FBS	fetal bovine serum
IC_50_	half-maximal inhibitory concentration
KR	β-ketoacyl reductase
LA	lyoniresinol-3α-*O*-β-arabinopyranoside
LR	lyoniresinol-3α-*O*-β-rhamnopyranoside
NADPH	nicotinamide adenine dinucleotide phosphate
